# Association of Race and Family Socioeconomic Status With Pediatric Postoperative Mortality

**DOI:** 10.1001/jamanetworkopen.2022.2989

**Published:** 2022-03-18

**Authors:** Brittany L. Willer, Christian Mpody, Joseph D. Tobias, Olubukola O. Nafiu

**Affiliations:** 1 Department of Anesthesiology and Pain Medicine, Nationwide Children’s Hospital, Columbus, Ohio; 2 College of Medicine, The Ohio State University, Columbus

## Abstract

**Importance:**

Racial disparities in postoperative outcomes have remained difficult to eliminate. It is commonly understood that socioeconomic status (SES) is an important factor associated with excess risk of postoperative morbidity and death. To date, comparable data exploring the association of family SES with pediatric postoperative mortality are unavailable, and it is unknown whether the advantage provided by higher income status is equitable across racial groups.

**Objective:**

To assess whether increasing family SES is associated with lower pediatric postoperative mortality and, if so, whether this association is equitable among Black and White children.

**Design, Setting, and Participants:**

This retrospective cohort study used data from 51 freestanding pediatric tertiary care hospitals across the US that reported to the Children’s Hospital Association Pediatric Health Information System. The study included 1 378 111 Black and White children younger than 18 years who underwent inpatient surgical procedures between January 1, 2004, and December 31, 2020.

**Exposures:**

The exposures of interest were race (Black and White) and parental income quartile (used as a proxy for SES and measured by median income quartile of the zip code of residence). Race was self-reported by parents or guardians at admission or assessed by the registration team consistent with each hospital’s policy and state legislation.

**Main Outcomes and Measures:**

The primary outcome was risk-adjusted in-hospital mortality rates by race and parental income quartile controlled for baseline covariates. To evaluate whether belonging to the highest income quartile modified the association between race and postoperative mortality, multiplicative and additive interactions were examined.

**Results:**

Among 1 378 111 children (773 364 [56.1%] male; mean [SD] age, 7 [6] years) who received inpatient surgical procedures during the study period, 248 464 children (18.0%) were Black, and 1 129 647 children (82.0%) were White; 211 127 children (15.3%) were Hispanic, and 825 477 (59.9%) were non-Hispanic. Only 49 541 Black children (20.3%) belonged to the highest income quartile compared with 482 758 White children (43.0%). The overall mortality rate was 1.2%, and mortality rates decreased as income quartile increased (1.4% in quartile 1 [lowest income], 1.3% in quartile 2, 1.0% in quartile 3, and 0.9% in quartile 4 [highest income]; *P* < .001). Among those belonging to the 3 lowest income quartiles, Black children had 33% higher odds of postoperative death compared with White children (adjusted odds ratio, 1.33; 95% CI, 1.27-1.39; *P* < .001). This racial disparity gap persisted among children belonging to the highest income quartile (adjusted odds ratio, 1.39; 95% CI, 1.25-1.54; *P* < .001). Postoperative mortality rates among Black children in the highest income quartile (1.30%; 95% CI, 1.19%-1.42%) were comparable to those of White children in the lowest income quartile (1.20%; 95% CI, 1.16%-1.25%). The interaction between Black race and income was not statistically significant on either the multiplicative scale (β for interaction = 1.04; 95% CI, 0.93-1.17; *P* = .45) or the additive scale (relative excess risk due to interaction = 0.01; 95% CI, −0.11 to 0.11; *P* > .99), suggesting no reduction in the disparity gap across increasing income levels.

**Conclusions and Relevance:**

In this cohort study, increasing SES was associated with lower pediatric postoperative mortality. However, postoperative mortality rates were significantly higher among Black children in the highest SES category compared with White children in the same category, and mortality rates among Black children in the highest SES category were comparable to those of White children in the lowest SES category. These findings suggest that increasing family SES did not provide equitable advantage to Black compared with White children, and interventions that target socioeconomic inequities alone may not fully address persistent racial disparities in pediatric postoperative mortality.

## Key Points

**Question:**

Is increasing socioeconomic status (SES) associated with lower pediatric postoperative mortality and, if so, is this association equitable among Black and White children?

**Findings:**

In this cohort study of 1 378 111 children who underwent inpatient operations in the US, increasing SES was associated with lower postoperative mortality. However, postoperative mortality rates were significantly higher among Black vs White children in the highest income quartile, and postoperative mortality rates among Black children in the highest income quartile were comparable to those of White children in the lowest income quartile.

**Meaning:**

In this study, substantial racial disparities in postoperative mortality across income quartiles suggest that increasing SES did not provide equitable advantage to Black children compared with White children.

## Teaser

This cohort study uses data from the Pediatric Health Information System to assess whether increasing family socioeconomic status is associated with lower pediatric postoperative mortality and, if so, whether this association is equitable among Black and White children.

## Introduction

Racial disparities in postoperative outcomes remain a problem despite considerable efforts to illuminate the scope of the issue and a recommendation by the Institute of Medicine to eliminate disparities in every area of health care.^1^ A study recently found that Black children were more likely than White children to die after unplanned reoperations.^2^ Even among healthy children, the risk-adjusted rates of postoperative morbidity and mortality were higher among Black children compared with their White peers.^3^ The underlying mechanisms associated with health disparities are unclear but are understandably complex and multifaceted. For example, the American Academy of Pediatrics reported that racial minority children have less access to primary care physicians and specialists and are more likely to have intervals longer than 1 year between physician visits.^4^ These findings translate to a higher risk of unmet health care needs among children with racial minority backgrounds; underdiagnosed and inadequately managed preoperative comorbid disease is a potential precursor of increased surgical complications.^5^

Beyond patient comorbidity burden, family socioeconomic status (SES) is another important source of health disparity.^6,7^ A monotonic association has been found between SES and health, with each increase in SES category associated with a stepwise improvement in an individual's health status.^1,8^ The converse also appears to be true, with decreasing SES associated with worse surgical outcomes.^9,10^ Notwithstanding the association between SES and health outcomes, it is challenging to precisely distinguish the consequences of SES for health status from those of race because race and SES are also associated. For instance, Black individuals disproportionately experience poverty and inequality compared with their White peers.^11,12^ Furthermore, racial minority children are more likely to be born into poverty and to persist at the bottom of the income distribution scale across generations.^13,14^ Thus, SES may mediate the association of race with health status, making it difficult to establish clear associations between these variables and surgical outcomes.

Moreover, the association between SES and health may not translate uniformly across racial groups. Whether high family SES provides equitable protection against postoperative morbidity and mortality among children of different racial backgrounds is presently unknown. Regardless of directionality, patient characteristics (such as SES and racial group) may have unequal consequences for postoperative morbidity or mortality. Therefore, the objective of this cohort study was to explore whether family SES is associated with lower pediatric postoperative mortality and, if so, to assess whether this association is equitable among Black and White children. We hypothesized that increasing family SES would be associated with reductions in the risk of pediatric postoperative death and that these reductions would vary between Black and White children.

## Methods

### Study Design and Population

We performed a retrospective analysis of the Children’s Hospital Association Pediatric Health Information System (PHIS).^57^ In brief, the PHIS is an administrative database that contains encounter-level data from 51 freestanding pediatric tertiary care hospitals across the US. These data are submitted by participating hospitals for external benchmarking purposes and include demographic information, diagnoses, procedures, billing codes for procedures, medications, and laboratory tests. Data veracity is ensured through a joint effort between the Children’s Hospital Association and participating hospitals. The institutional review board of Nationwide Children’s Hospital approved the study protocol, with a waiver of informed consent granted because data were deidentified. This study followed the Strengthening the Reporting of Observational Studies in Epidemiology (STROBE) reporting guideline for cohort studies.

We queried the PHIS database for children of Black and White races who were younger than 18 years and underwent inpatient surgical procedures between January 1, 2004, and December 31, 2020. We decided a priori to focus on Black and White children to build on findings from previous studies^2,3,37,61^ investigating racial disparities in postoperative adverse events, perioperative pain management, and surgical mortality risk. We excluded patients with missing information on race and median household income of the zip code of residence.

### Variable Definitions

Our primary outcome was in-hospital postoperative mortality, defined as death after an index surgical procedure. We used household income as a proxy for family SES, with income based on the median household income of the patient's zip code of residence as estimated for 2015. We grouped household income into 4 quartiles, with quartile 1 (lowest income) indicating less than $33 190 per year, quartile 2 indicating $33 190 to $41 850 per year, quartile 3 indicating $41 851 to $54 728 per year, and quartile 4 (highest income) indicating more than $54 728 per year. Race was self-reported by the parents or guardians at admission or assessed by the registration team consistent with each hospital’s policy and state legislation. For risk-adjusted mortality rates, we adjusted for age (<12 months, 12 months to 5 years, 6-12 years, or >12 years), sex (male vs female), ethnicity (Hispanic vs non-Hispanic), insurance (commercial, Medicaid, other, or none), hospital census region (Midwest, Northeast, South, or West), 8 preoperative complex chronic conditions (each coded as a binary variable, including cardiovascular, gastrointestinal, hematologic or immunologic, cancer, metabolic, neurological and neuromuscular, kidney and urological, or respiratory conditions), and 9 surgical procedure groups (cardiovascular, digestive, hematologic or oncological, neonatological, neurological, orthopedic or joint disease, respiratory, transplant, or other procedures). Surgical procedure groups, derived from PHIS service line data, were created by analyzing the All-Patient Refined Diagnosis Related Groups,^58^ Centers for Medicare & Medicaid Services Diagnosis Related Groups,^59^ and Medicare Severity Diagnosis Related Groups^60^ systems. The Centers for Medicare & Medicaid Services Diagnosis Related Groups classification system uses several factors along with principal diagnosis and surgical procedure to categorize patients into homogeneous groups according to severity of illness, complexity of procedures, and resource use. Complex chronic conditions were defined according to Feudtner et al as “any medical condition that can be reasonably expected to last at least 12 months (unless death intervenes) and to involve either several different organ systems or 1 organ system severely enough to require specialty pediatric care and probably some period of hospitalization in a tertiary care center.”^15^^(p199)^

### Statistical Analysis

We compared baseline characteristics of White and Black children. To calculate risk-adjusted in-hospital mortality rates by race and household income categories, we fitted a logistic regression model (details available in eMethods in the Supplement). We then used the margin postestimation command in Stata software, version 16.1 (StataCorp LLC), to estimate risk-adjusted mortality rates and 95% CIs.

To evaluate whether highest household income quartile status modified the association between race and the risk of postoperative death, we fitted a multivariable logistic regression model including a 2-way interaction between race and median income of the zip code of residence. The zip code of residence was used as a binary variable to indicate whether patients belonged to the highest income quartile.^16^

Results were reported as odds ratios (ORs) with corresponding 95% CIs. All analyses were performed using Stata software, version 16 (StataCorp LLC). The significance threshold was 2-tailed *P* < .05.

## Results

### Characteristics of Study Population

We identified 1 378 111 children (mean [SD] age, 7 [6] years) who received inpatient surgical procedures at PHIS-participating centers between January 1, 2004, and December 31, 2020. Of those, 248 464 children (18.0%) were Black, and 1 129 647 children (82.0%) were White. Overall, 604 747 children (43.9%) were female, 773 364 (56.1%) were male, 211 127 (15.3%) were Hispanic, and 825 447 (59.9%) were non-Hispanic. Only 49 541 Black children (20.3%) belonged to the highest quartile of parental income for zip code compared with 482 758 White children (43.0%). White children were more likely to have commercial insurance (28.2%) than Black children (23.8%) (Table 1).

**Table 1.** Characteristics of Study Population^a^

Characteristic	Participants, No. (%)		
	Overall (N = 1 378 111)	Black race (n = 248 464)	White race (n = 1 129 647)
Sex			
Female	604 747 (43.9)	106 794 (43.0)	497 953 (44.1)
Male	773 364 (56.1)	141 670 (57.0)	631 694 (55.9)
Ethnicity			
Hispanic	211 127 (15.3)	6156 (2.5)	204 971 (18.1)
Non-Hispanic	825 477 (59.9)	173 237 (69.7)	652 240 (57.7)
Unknown	341 507 (24.8)	69 071 (27.8)	272 436 (24.1)
Age			
Adolescent (>12 y)	319 079 (23.2)	58 085 (23.4)	260 994 (23.1)
Child (6-12 y)	379 713 (27.6)	63 511 (25.6)	316 202 (28.0)
Young child (12 mo-5 y)	339 968 (24.7)	63 243 (25.5)	276 725 (24.5)
Infant (<12 mo)	339 351 (24.6)	63 625 (25.6)	275 726 (24.4)
Household income quartile of zip code of residence^b^			
1 (lowest)	21 596 (1.6)	3428 (1.4)	18 168 (1.6)
2	222 910 (16.3)	27 649 (11.3)	195 261 (17.4)
3	589 100 (43.1)	163 775 (67.0)	425 325 (37.9)
4 (highest)	532 299 (39.0)	49 541 (20.3)	482 758 (43.0)
Unknown	12 206 (0.9)	4071 (1.6)	8135 (0.7)
Insurance			
Commercial	377 667 (27.4)	59 049 (23.8)	318 618 (28.2)
Medicaid	193 255 (14.0)	33 680 (13.6)	159 575 (14.1)
Other	495 650 (36.0)	133 471 (53.7)	362 179 (32.1)
None	311 539 (22.6)	22 264 (9.0)	289 275 (25.6)
Hospital census region			
Midwest	344 720 (25.0)	99 997 (40.2)	244 723 (21.7)
Northeast	344 338 (25.0)	61 554 (24.8)	282 784 (25.0)
South	344 827 (25.0)	51 423 (20.7)	293 404 (26.0)
West	344 226 (25.0)	35 490 (14.3)	308 736 (27.3)
Elective vs nonelective case			
Elective	643 494 (46.7)	122 733 (49.4)	520 761 (46.1)
Nonelective	510 162 (37.0)	89 798 (36.1)	420 364 (37.2)
Unknown	224 455 (16.3)	35 933 (14.5)	188 522 (16.7)
Preoperative complex chronic conditions			
Cardiovascular	215 765 (15.7)	40 959 (16.5)	174 806 (15.5)
Gastrointestinal	216 307 (15.7)	42 681 (17.2)	173 626 (15.4)
Hematological or immunological	48 730 (3.5)	18 244 (7.3)	30 486 (2.7)
Cancer	84 757 (6.2)	12 742 (5.1)	72 015 (6.4)
Metabolic	62 819 (4.6)	12 395 (5.0)	50 424 (4.5)
Neurological and neuromuscular	248 953 (18.1)	45 244 (18.2)	203 709 (18.0)
Kidney and urological	134 282 (9.7)	19 294 (7.8)	114 988 (10.2)
Respiratory	91 303 (6.6)	19 833 (8.0)	71 470 (6.3)
Procedural group			
Cardiovascular	115 803 (8.4)	20 639 (8.3)	95 164 (8.4)
Digestive	279 267 (20.3)	40 017 (16.1)	239 250 (21.2)
Hematological and oncological	22 944 (1.7)	5161 (2.1)	17 783 (1.6)
Neonatological	79 750 (5.8)	15 894 (6.4)	63 856 (5.7)
Neurological	164 182 (11.9)	27 318 (11.0)	136 864 (12.1)
Orthopedic and joint disease	252 064 (18.3)	48 729 (19.6)	203 335 (18.0)
Other	405 386 (29.4)	80 901 (32.6)	324 485 (28.7)
Respiratory	40 371 (2.9)	6281 (2.5)	34 090 (3.0)
Transplant	18 344 (1.3)	3524 (1.4)	14 820 (1.3)

^a^ Among Black and White children recorded in the Children’s Hospital Association Pediatric Health Information System who received inpatient surgical procedures between January 1, 2004, and December 31, 2020.

^b^ Quartile 1 indicates less than $33 190 per year; quartile 2, $33 190 to $41 850 per year; quartile 3, $41 851 to $54 728 per year; and quartile 4 (highest income), more than $54 728 per year.

### Overall Mortality by Parental Income

The overall postoperative mortality rate for the study cohort was 1.2% (15 967 patients). Mortality rates decreased as parental income quartile increased (4865 [1.4%] in quartile 1 [lowest income], 4400 [1.3%] in quartile 2, 3737 [1.0%] in quartile 3, and 2965 [0.9%] in quartile 4 [highest income]; *P* < .001).

### Race-Specific Mortality by Parental Income

Postoperative mortality rates varied significantly by race, with a higher proportion of deaths occurring among Black children (4102 of 248 464 patients [1.7%]) compared with White children (11 865 of 1 129 647 patients [1.1%]). A difference in the advantage provided by higher income status was found across racial groups, with lower postoperative mortality associated with White race compared with Black race. For example, among Black children, the mortality rate difference between those in the highest vs lowest quartile of parental income was smaller (1.30% [95% CI, 1.19%-1.42%] vs 1.50% [95% CI, 1.43%-1.57%]; adjusted OR [aOR], 0.86; 95% CI, 0.77-0.96; *P* = .005) than the difference among White children in those categories (0.96% [95% CI, 0.93%-1.00%] vs 1.20% [95% CI, 1.16%-1.25%]; aOR, 0.78; 95% CI, 0.74-0.83; *P* = .002) (eTable in the Supplement). Postoperative mortality rates among Black children with parents in the highest income quartile (1.30%) were comparable to those of White children with parents in the lowest income quartile (1.20%) (Figure).

**Figure.**
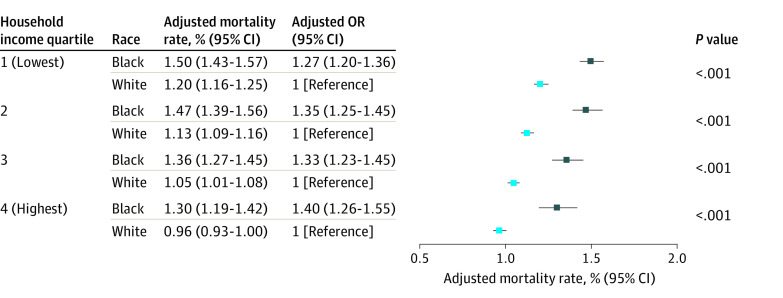
Adjusted Incidence of Inpatient Death Across Increasing Levels of Household Income by Zip Code Based on data from Black and White children recorded in the Children’s Hospital Association Pediatric Health Information System who received inpatient surgical procedures between January 1, 2004, and December 31, 2020. Adjusted mortality rates were controlled for sex, Hispanic ethnicity, age, insurance status, complex chronic conditions, and procedural group. Details about the model are available in eMethods in the Supplement. OR indicates odds ratio.

### Racial Disparity Across Increasing Levels of Income

Among children belonging to the 3 lowest quartiles of median parental income based on zip code, Black race was associated with 33% greater odds of postoperative death compared with White race (aOR, 1.33; 95% CI, 1.27-1.39; *P* < .001). The disparity gap persisted among children belonging to the highest parental income quartile (aOR, 1.39; 95% CI, 1.25-1.54; *P* < .001). The interaction between Black race and income was not statistically significant on either the multiplicative scale (β for interaction = 1.04; 95% CI, 0.93-1.17; *P* = .45) or the additive scale (relative excess risk due to interaction = 0.01; 95% CI, −0.11 to 0.11; *P* > .99), suggesting no reduction in the disparity gap across increasing levels of income (Table 2). We also did not find a significant interaction between Black race and income when stratifying patients by intensive care unit bed use (multiplicative scale: β for interaction = 1.02 [95% CI, 0.85-1.24; *P* = .81]; additive scale: relative excess risk due to interaction = −0.04 [95% CI, −0.23 to 0.14; *P* = .65]).

**Table 2.** Race-Specific Incidence of Inpatient Death Across Increasing Levels of Household Income^a^

Race	Household income quartile				Interaction			
	1-3		4		Multiplicative scale		Additive scale	
	OR (95% CI)	*P* value	OR (95% CI)	*P* value	β (95% CI)	*P* value	RERI (95% CI)	*P* value
Overall (N = 1 378 111)								
Black	1.33 (1.27 to 1.39)	<.001	1.39 (1.25 to 1.54)	<.001	1.04 (0.93 to 1.17)	.45	0.01 (−0.11 to 0.11)	>.99
White	1 [Reference]		1 [Reference]		1 [Reference]		1 [Reference]	
No ICU bed use (n = 962 930)								
Black	1.12 (1.06 to 1.18)	<.001	1.22 (1.07 to 1.39)	<.001	1.09 (0.95 to 1.25)	.22	0.07 (−0.06 to 0.20)	.32
White	1 [Reference]		1 [Reference]		1 [Reference]		1 [Reference]	
ICU bed use (n = 415 181)								
Black	1.66 (1.55 to 1.78)	<.001	1.70 (1.42 to 2.04)	<.001	1.02 (0.85 to 1.24)	.81	−0.04 (−0.23 to 0.14)	.65
White	1 [Reference]		1 [Reference]		1 [Reference]		1 [Reference]	

Abbreviations: ICU, intensive care unit; OR, odds ratio; RERI, relative excess risk due to interaction.

^a^ Household income was based on the median income of the patient’s zip code of residence. Details of the model are available in eMethods in the Supplement.

## Discussion

This cohort study examined whether and to what extent family SES was associated with pediatric postoperative in-hospital mortality and whether postoperative mortality differed across racial groups. Among both Black and White children, there was a gradual decrease in the risk of postoperative death as the household income of the patient’s zip code increased. However, we found no data suggesting that increasing income levels were associated with decreases in the racial disparity gap, on either the multiplicative or additive scale. In addition, there were substantial differences across racial groups with regard to the advantage provided by higher income status, with White children experiencing lower postoperative mortality rates than Black children in the same income quartile. Among Black children, the mortality rate difference between those in the highest vs lowest quartiles of parental income was smaller than that of White children in the highest vs lowest quartiles. Postoperative mortality rates among Black children with parents in the highest income quartile were comparable to those of White children with parents in the lowest income quartile.

Despite substantial improvements in pediatric perioperative care within the last 3 decades, disparity in surgical outcomes has persisted, with Black children having higher rates of postoperative complications and mortality than their White peers.^17,18,19,20,21,22,23,24,25,26,27,28,29,30,31,32,33,34^ Traditional explanations for these racial disparities include greater preoperative comorbidity burden among Black children, higher likelihood that Black children will receive treatment in low-performing hospitals, and higher likelihood that Black families will have lower SES.^17,34,35,36,37,38^

An association between lower SES and worse health outcomes has been previously reported. Several investigators have found a monotonic association between SES and various health measures, including postoperative morbidity and mortality.^39,40^ Worse outcomes among patients with lower SES have been reported for a variety of procedures, including cholecystectomy^41^ and oncological,^42^ cardiac,^43^ brain tumor,^44^ and carotid artery^45^ procedures. Our finding that the highest postoperative mortality occurred among both Black and White children in the lowest SES quartile was consistent with the findings of these earlier studies.^39,40,41,42,43,44,45^ A common factor among children who died after surgical procedures was not solely their race but their family’s SES. However, the association between family SES and postoperative mortality does not preclude the impact of race as a factor; our analysis suggested that the protection against postoperative death provided by family SES was not equitable across Black vs White race.

The present study is a novel addition to the literature because it evaluated the association of race with SES and the consequences of this association for pediatric postoperative surgical outcomes. We assessed the interaction between race and SES by calculating the relative excess risk due to the association between race and median parental income by zip code of residence, which was a more appropriate and nuanced method than covariate adjustment. Our findings suggest that a universal solution to pediatric postoperative death may not be attainable. For example, although hospital-based clinical interventions addressing traditional risk factors may be associated with reductions in postoperative complications and mortality, they are unlikely to eliminate disparities in surgical outcomes without directly addressing upstream factors associated with low SES.

Our observation that the survival advantage of belonging to the highest income quartile was more substantial among White children than Black children was consistent with a previous report^8^ involving adult patients, which suggested diminishing returns for upward mobility among financially successful Black families compared with their White peers. Although a health advantage has been found among White patients with higher SES, the same advantage may not always be observed among racial minority patients. The diminishing returns principle encapsulates this issue, suggesting that gains in the factors used to construct SES (educational, occupational, and/or income attainment) may not translate to improved health among racial minority individuals.^8^ Farmer and Ferraro^8^ examined the association between income and health using data from the US National Health and Nutrition Examination Survey, with follow-up interviews conducted over 20 years. The researchers found that as income levels increased, self-rated health improved faster among White adults than Black adults, with Black adults at the highest income levels reporting substantially lower self-rated health than their White peers.^8^ In addition, they found that Black adults with the highest occupational prestige reported worse self-rated health than their White counterparts and, as educational levels increased, self-rated health improved among White adults only.^8^

Why our analysis of in-hospital postoperative mortality was consistent with population-level adult data regarding racial inequality in the advantage provided by higher income status is unclear. It is concerning that postoperative mortality rates among Black children in the highest income quartile were not only higher than those of their White peers in the same group but were comparable to rates among White children in the lowest income quartile. Our findings of disparate mortality risk by race and SES did not appear to differ by processes of care; no significant interaction was found between Black race and income among patients requiring vs not requiring ICU beds. Our findings suggest that measures to reduce racial disparity in pediatric postoperative mortality must look beyond social factors, such as family SES or preoperative comorbidity burden. Examination of hospital systems to assess variability in health care intensity and quality of outcomes is warranted. The quality of care in hospital systems may be easier to address than patient-level variables, such as SES and disease burden at presentation for surgical procedures. Furthermore, consideration of additional actions by hospital systems to reduce medical debt for disadvantaged patients, such as those with low SES and/or those belonging to minority groups, is warranted.

### Limitations

This study has limitations. We relied on zip code–level median household income as a proxy for family SES. We recognize that SES is a multidimensional construct that involves many aspects of a child’s environment, and it is a crude measure of individual-level financial status or income-to-need ratio. Nonetheless, zip code–level median household income has been used by previous investigators as a broad indicator of SES.^46^ Furthermore, other important measures of SES, such as income inequality, neighborhood deprivation, structural racism, and adverse environmental conditions, appear to cluster in geographic areas.^47^ In addition, SES is affected by educational level, occupation, and other unmeasurable factors. The use of income as the sole indicator of SES means that our analysis did not account for the possibility of status incongruence or status inconsistency, concepts that describe the state of individuals who simultaneously hold positions of unequal societal rank (ie, high educational attainment but low-status job or low educational attainment but high income).^48^ Children of these individuals may not have been included in the analyses of SES groups to which social comparisons are typically ascribed.

Our study provides a national picture of the association of family SES with racial disparities in pediatric postoperative mortality. However, inequities in health care delivery and outcomes have been reported both between and within hospitals.^49^ The present study is unable to evaluate these disparities on an institutional level. In addition, multiracial children are difficult to describe in large administrative databases, which are typically limited to categorical race definitions. In 2015, almost 15% of infants in the US could be considered multiracial,^50^ but fewer than 40% of multiracial adults considered themselves as such.^51^ Because race is designated by parental self-report (hence based on parental racial identity), this study is unlikely to fully account for the multiracial nature of the included patients. However, given that the typical practice is to assign a child’s race at birth based on the racial identity of the non-White parent, especially in the case of parents who are Black and White and the understanding that race is not a biological but a social construct,^52,53^ we do not believe our findings would be invalidated by the fact that we did not fully account for the consequences of multiracial categorization. We acknowledge that we could not evaluate the role of additional processes of care, including do-not-resuscitate status, which has been found to differ across populations by race.^54^ In addition, the database used for the present study did not contain granular information about variation in surgical profiles (ie, specialty, complexity, and postoperative complications) across racial groups, which limits our ability to add clinical context to the findings. Although we do not have any way to ascribe structural racism to our findings, some investigators have suggested that systemic discrimination, economic and spatial segregation and deprivation, and social factors associated with health are likely to explain the worse health outcomes observed among racial minority pediatric patients.^7,55,56^

## Conclusions

Using a nationally representative administrative database, this cohort study found that the interaction between family SES and race was significantly associated with disparities in pediatric postoperative mortality. The findings also suggested that there were disparities in the relative protection against postoperative death provided by increasing family SES to the extent that the advantage associated with higher income status did not accrue equally to Black vs White children. Thus, interventions addressing socioeconomic disparities alone may not fully address persistent racial disparities in pediatric postoperative mortality. A multifaceted approach that includes dismantling of socioeconomic barriers, equitable availability of comprehensive pediatric surgical care, and personalized care for children of all races is needed.

## Notes

Supplement. **eMethods.** Logistic Regression Models **eTable.** Mortality Benefits Associated With Increasing Income by Race
