# Supplementary material for: Association of Race and Family Socioeconomic Status With Pediatric Postoperative Mortality
Source: JAMA Netw Open. 2022 Mar 18;5(3):e222989. doi: 10.1001/jamanetworkopen.2022.2989 (PMC8933731; doi:10.1001/jamanetworkopen.2022.2989)
Supplement: Supplement. — eMethods. Logistic Regression Models eTable. Mortality Benefits Associated With Increasing Income by Race [file jamanetwopen-e222989-s001.pdf]

Supplementary Online Content

Willer BL, Mpody C, Tobias JD, Nafiu OO. Association of race and family socioeconomic status with pediatric postoperative mortality. *JAMA Netw Open*. 2022;5(3):e222989. doi:10.1001/jamanetworkopen.2022.2989

eMethods. Logistic Regression Models

eTable. Mortality Benefits Associated With Increasing Income by Race

This supplementary material has been provided by the authors to give readers additional information about their work.

© 2022 Willer BL et al. *JAMA Network Open*.

eMethods. Logistic Regression Models

The following logistic regression model was fit to calculate risk-adjusted in-hospital mortality rates by race and household income categories:

$\text{Log[odds}(Y_i)] = \beta_o + \beta_1 \text{black}_i + \beta_2 \text{income\_Q2} + \beta_3 \text{income\_Q3} + \beta_4 \text{income\_Q3} + \beta_5 \text{black}_i \times \text{income\_Q2} + \beta_6 \text{black}_i \times \text{income\_Q3} + \beta_7 \text{black}_i \times \text{income\_Q4} + \beta X$, where Y_i is whether patient *ith* died during the hospitalization, black is coded as 1 = the patient is of Black race, and 0 = the patient is of White race; income Q2 is coded as 1 = the patient belongs to the second quartile of income, and 0 = otherwise; income Q3 is coded as 1 = the patient belongs to the third quartile of income, and 0 = otherwise; income Q4 is coded as 1 = the patient belongs to the fourth quartile [highest] of income, and 0 = otherwise; and X is the design matrix representing other covariates.

To evaluate whether belonging to the highest household income quartile modified the risk of postoperative mortality associated with race, we fitted a multivariable logistic regression model including a two-way interaction between race and income for ZIP Code, operationalized as a binary indicator of whether patients belonged to the highest quartile of income categories. The following model was fit:

$\text{Log[odds}(Y_i)] = \beta_o + \beta_1 \text{black}_i + \beta_2 \text{income\_Q4} + \beta_3 \text{black}_i \times \text{income\_Q4} + \beta X$, where income Q4 is coded as 1 = the patient belongs to the fourth quartile [highest] for the income and 0 = otherwise. This resulted in the following estimates: β_1 is the log(OR) of mortality, comparing Black to White children, within the first three quartiles of income; ($\beta_1 + \beta_3$) is the log(OR) of mortality comparing Black to White children, within the highest quartile of income; β_3 is the multiplicative interaction between race and income; and the relative excess risk due to interaction (RERI) is $\exp(\beta_1 + \beta_2 + \beta_3) - \exp(\beta_1) - \exp(\beta_2) + 1$

For data shown in Table 2 and the Figure, the following model was fit to calculate the race-specific incidence of inpatient mortality across increasing levels of household income by zip code:

$\text{Log}(E[Y_i]) = \beta_o + \beta_1 \text{black}_i + \beta_2 \text{income\_Q4} + \beta_3 \text{black}_i \times \text{income\_Q4} + \beta X$, where Y_i is whether patient *ith* died during the hospitalization, black is code as 1 the patient is of Black race and 0 if the patient is of White race; income_Q4 is coded 1 if the patient belongs to the fourth quartile (highest) for the income and 0 if otherwise; and X is the design matrix representing covariates, including age, sex, ethnicity, insurance, hospital census region, and 8 different preoperative complex chronic conditions (cardiovascular, gastrointestinal, hematologic or immunologic, malignancy, metabolic, neurologic and neuromuscular, kidney and urologic, and respiratory), each coded as binary variable, and procedural group (cardiovascular, digestive, hematology/oncology, neonatology, neurology, orthopedics/joint disease, other, respiratory, and transplant). β_1 is the log(OR) of mortality comparing Black with White children within the first 3 income quartiles. $\beta_1 + \beta_3$ is the log(OR) of mortality comparing Black with White children within the highest quartile (4) of income. β_3 is the multiplicative interaction between race and income. RERI was calculated as $\exp(\beta_1 + \beta_2 + \beta_3) - \exp(\beta_1) - \exp(\beta_2) + 1$.

© 2022 Willer BL et al. *JAMA Network Open*.

eTable. Mortality Benefits Associated With Increasing Income by Race

Race	Household income	Adjusted mortality rate, in % (95%CI)*	Adjusted odds ratio (95%CI)*	P-value
Black	First quartile (Lowest)	1.50(1.43,1.57)	Reference	
	Second quartile	1.47(1.39,1.56)	0.98(0.91,1.07)	0.005
	Third quartile	1.36(1.27,1.45)	0.90(0.82,0.98)	<0.001
	Fourth quartile (Highest)	1.30(1.19,1.42)	0.86(0.77,0.96)	<0.002
White	First quartile (Lowest)	1.20(1.16,1.25)	Reference	
	Second quartile	1.13(1.09,1.16)	0.93(0.88,0.98)	0.68
	Third quartile	1.05(1.01,1.08)	0.86(0.81,0.90)	0.02
	Fourth quartile (Highest)	0.96(0.93,1.00)	0.78(0.74,0.83)	0.005

© 2022 Willer BL et al. *JAMA Network Open*.
